# Travel-related infections presenting in Europe: A 20-year analysis of EuroTravNet surveillance data

**DOI:** 10.1016/j.lanepe.2020.100001

**Published:** 2020-11-12

**Authors:** Martin P. Grobusch, Leisa Weld, Abraham Goorhuis, Davidson H. Hamer, Mirjam Schunk, Sabine Jordan, Frank P. Mockenhaupt, François Chappuis, Hilmir Asgeirsson, Eric Caumes, Mogens Jensenius, Perry J.J. van Genderen, Francesco Castelli, Rogelio López-Velez, Vanessa Field, Emmanuel Bottieau, Israel Molina, Christophe Rapp, Marta Díaz Ménendez, Effrossyni Gkrania-Klotsas, Carsten S. Larsen, Denis Malvy, David Lalloo, Federico Gobbi, Simin A. Florescu, Philippe Gautret, Patricia Schlagenhauf, for EuroTravNet

**Affiliations:** a Centre of Tropical Medicine and Travel Medicine, Amsterdam University Medical Centres, location AMC, Amsterdam Infection & Immunity, Amsterdam Public Health, Amsterdam, the Netherlands; b Statistical Consultant, Geneva, Switzerland; c Department of Global Health, Boston University School of Public Health and Section of Infectious Diseases, Department of Medicine, Boston Medical Center, Boston, MA, USA; d Department of Infectious Diseases and Tropical Medicine (DITM), Ludwig-Maximilians University of Munich, Munich, Germany; e Division of Tropical Medicine, I. Department of Medicine, University Medical Center Hamburg-Eppendorf & Department of Tropical Medicine, Bernhard Nocht Institute for Tropical Medicine, Hamburg, Germany; f Charité-Universitätsmedizin Berlin, corporate member of Freie Universität Berlin, Humboldt-Universität Berlin & Berlin Institute of Health, Institute of Tropical Medicine and International Health, Berlin, Germany; g Division of Tropical and Humanitarian Medicine, Geneva University Hospitals, Geneva, Switzerland; h Department of Infectious Diseases, Karolinska University Hospital, Stockholm, Sweden & Unit of Infectious Diseases, Department of Medicine, Huddinge, Karolinska Institutet, Stockholm, Sweden; i Sorbonne Université, INSERM, Institut Pierre Louis d’Épidémiologie et de Santé Publique (IPLESP), AP-HP, Hôpitaux Universitaires Pitié-Salpêtrière Charles Foix, Service de Maladies Infectieuses et Tropicales, Paris, France; j Department of Infectious Diseases, Oslo University Hospital, Ullevål, Oslo, Norway; k Department of Microbiology and Infectious Diseases, University Hospital Erasmus MC, Rotterdam, the Netherlands; l University of Brescia & ASST Spedali Civili de Brescia, Brescia, Italy; m National Referral Unit for Tropical Diseases, Infectious Diseases Department, Ramón y Cajal University Hospital Madrid, Madrid, Spain; n InterHealth Worldwide, Newington Causeway, London, United Kingdom; o Institute of Tropical Medicine, Antwerp, Belgium; p Department of Infectious Diseases, Vall d'Hebron University Hospital, PROSICS Barcelona, Barcelona, Spain; q Infectious and Tropical Diseases Department, HIA Bégin, Saint-Mandé, France; r National Referral Unit for Imported Tropical Diseases, Department of Internal Medicine, Hospital Universitario La Paz-Carlos III, IdiPAZ, Madrid, Spain; s Department of Medicine, University of Cambridge, Cambridge, United Kingdom; t Department of Infectious Diseases, Aarhus University Hospital, Aarhus, Denmark; u Unit of Tropical Medicine and Clinical International Health, Department of Infectious and Tropical Diseases, University Hospital Center of Bordeaux & INSERM 1219, University of Bordeaux, Bordeaux, France; v Liverpool School of Tropical Medicine and Tropical Infectious Disease Unit, Royal Liverpool University Hospital, Liverpool, United Kingdom; w Department of Infectious/Tropical Diseases and Microbiology, IRCCS Sacro Cuore Don Calabria Hospital, Negrar, Verona, Italy; x Dr. Victor Babes Clinical Hospital of Infectious and Tropical Diseases, Bucharest, Romania; y University Hospital Institute Méditerranée Infection, Aix-Marseille Univ, IRD, AP-HM, SSA, VITROME, Marseille, France; z University of Zürich Centre for Travel Medicine, WHO Collaborating Centre for Travellers’ Health, Department of Public Health, Institute for Epidemiology, Biostatistics and Prevention, Zürich, Switzerland

**Keywords:** Eurotravnet, Geosentinel, Infectious diseases, Sentinel surveillance, travel

## Abstract

**Background:**

Disease epidemiology of (re-)emerging infectious diseases is changing rapidly, rendering surveillance of travel-associated illness important.

**Methods:**

We evaluated travel-related illness encountered at EuroTravNet clinics, the European surveillance sub-network of GeoSentinel, between March 1, 1998 and March 31, 2018.

**Findings:**

103,739 ill travellers were evaluated, including 11,239 (10.8%) migrants, 89,620 (86.4%) patients seen post-travel, and 2,880 (2.8%) during and after travel. Despite increasing numbers of patient encounters over 20 years, the regions of exposure by year of clinic visits have remained stable. In 5-year increments, greater proportions of patients were migrants or visiting friends and relatives (VFR); business travel-associated illness remained stable; tourism-related illness decreased. Falciparum malaria was amongst the most-frequently diagnosed illnesses with 5,254 cases (5.1% of all patients) and the most-frequent cause of death (risk ratio versus all other illnesses 2.5:1). Animal exposures requiring rabies post-exposure prophylaxis increased from 0.7% (1998–2002) to 3.6% (2013–2018). The proportion of patients with seasonal influenza increased from zero in 1998–2002 to 0.9% in 2013–2018. There were 44 cases of viral haemorrhagic fever, most during the past five years. Arboviral infection numbers increased significantly as did the range of presenting arboviral diseases, dengue and chikungunya diagnoses increased by 2.6% and 1%, respectively.

**Interpretation:**

Travel medicine must adapt to serve the changing profile of travellers, with an increase in migrants and persons visiting relatives and friends and the strong emergence of vector-borne diseases, with potential for further local transmission in Europe.

**Funding:**

This project was supported by a cooperative agreement (U50CK00189) between the Centers for Disease Control and Prevention to the International Society of Travel Medicine (ISTM) and funding from the ISTM and the Public Health Agency of Canada.

**Research in Context**

**Evidence before this study**

Over the past 20 years, sentinel surveillance has emerged as a valuable tool to record and analyse trends in travellers importing infectious diseases from endemic areas to non-endemic areas, providing insight in changing infectious diseases epidemiology worldwide and assisting physicians in making diagnostic and therapeutic decisions when caring for returning tourists and migrants.

**Added value of this study**

The dataset and analysis presented here is the most comprehensive published to date on changing trends over time in travel medicine, focusing on infectious diseases imported into Europe.

**Implications of all the available evidence**

Detailed sentinel surveillance data contribute to recognising emerging and changing infectious diseases trends, and inform decision-making in pre- and post-travel care.

Alt-text: Unlabelled box

## 1 Introduction

The global population currently encompasses more than 7.6 billion people, growing at annual rates between 5 and 10% [1]. The mobility within this population is well illustrated with 609 million international tourist arrivals in 1998 and 1408 in 2018, an increase of 130% [2], with a constant upward future trend, particularly across emerging economies [3]. At the same time, never have more people been displaced: an estimated 68.5 million, with more than 25 million refugees worldwide; the majority of whom are under-age minors [4]. This means that the prevention and treatment of illness associated with human mobility has become an increasingly important topic.

The International Society of Travel Medicine's (ISTM) [5] European Travel and Tropical Medicine Network (EuroTravNet) [6] is a sub-network of the global GeoSentinel Surveillance Network [7], and currently includes 25 core sites. These are large-volume pre- and post-travel academic or clinical settings in twelve predominantly Western European countries [6]. The network captures travel-related illness in returning travellers and migrants as part of its portfolio. It contributes to detection, identification and management of imported infections, detection of outbreaks, and characterization of trends in travel-related illness over time [8,9].

Here, we provide a 20-year analysis of travel-related illness presenting at EuroTravNet clinics and propose up-to-date priorities in travel medicine practice based on these trends.

## 2 Methods

Detailed methods for patient recruitment, inclusion criteria, diagnosis tests and limitations of the EuroTravNet and GeoSentinel databases have been described elsewhere [9,10]. In brief, patients must have crossed an international border within the last 12 months before the clinic visit, and must have sought medical care for a presumed travel-related illness, both infectious or non-infectious, or for screening for asymptomatic infection. All travellers presenting to one of the EuroTravNet sites are systematically included in the GeoSentinel database. The diagnosis is based on specific clinical and/or laboratory diagnostic criteria for each infectious disease that are harmonised across sites through the use of a common set of specific definitions for each infection caused by a specific pathogen, or based on an infectious syndrome (with syndromic codes used when a clinical diagnosis is made and no specific etiology is identified) [9]. The data collection protocol is classified as public health surveillance and not human subjects research, by the institutional review board officer at the United States National Center for Emerging and Zoonotic Infectious Diseases, at the Centers for Disease Control and Prevention (CDC).

Travellers who presented between March 1, 1998 and March 31, 2018 to a EuroTravNet site with a travel-related illness were included. We evaluated two different clinical settings; patients who sought care during or after travel; and travellers whose only travel purpose was migration and were seen by a clinician after arrival in their destination country (referred to as migrants). Patients without a diagnosis yet, who were found to be healthy, or whose illness was not travel-related or the diagnosis was neither confirmed nor probable, were excluded. Diagnoses were analysed as a proportion of patients to control for the growth in number of patients seen over time. Because such a large sample size can result in statistically significant but clinically non-important differences and because denominator data to calculate risks is not available, we have limited statistical significance testing except to this very specific question of whether the proportion of patients with a disease increased or decreased over time. Grouping years into 5-year intervals was introduced to reduce year-to-year variability.

The chi-square test was used to determine whether there is an association between any variable and the year of clinic visit (grouped into 5-year intervals: 1998–2002, 2003–2007, 2008–2012 and 2013–2018). This analysis focuses on infectious diseases reports, constituting the vast majority of EuroTravNet records. Although the number of clinics in the network has increased, they continue to be large-volume pre- and post-travel academic or clinical settings. Because the number of clinics and patients has grown over time, all analyses over time examine the number of diagnoses as a proportion of the diagnoses reported in the same time period. We look at the number of diagnoses and the number as a proportion of the ill patients seen to analyse whether the amount of a disease has changed as a percentage of the patients we see. If the test was significant, the standardised residuals were examined to determine the largest contributions to the lack of independence. If the chi square test was significant and the association appeared monotonically increasing or decreasing, Somers’ D was used to test that hypothesis. Additional sensitivity analyses were performed based on only five clinics that joined the network in the first six years to assess the possibility that trends were due to the inclusion of new clinics. Data were analysed using Stata 14.2 for Windows (StataCorp LP, College Station, TX, USA).

### 2.1 Role of the funding source

The funders had no role in study design, data collection, data analysis, interpretation, and writing of the report.

## 3 Results

### 3.1 Cohort characteristics

From March 1998 to March 2018, 25 centers in 13 countries contributed 119,826 patient records. Over 20 years, the number of clinics participating in the network has grown. Five clinics joined early and others along the way; with a total of 25 having contributed data over the years. The number of patients reported has increased from 6267 in 1998–2002 to 51,162 during 2013–2018. After exclusion of 16,087 records, 103,739 ill travellers were included in the analysis (Suppl. Table 1). Most (92,500/103,739; 89.2%) ill travellers were seen during or post-travel.

There is a significant association between age group and the year of clinic visit (*p*<.0001). There was an increase over time in the proportion of patients who were aged 10–20 (3% to 7%) and who were aged over 50 (17% to23%) and a decrease in the proportion of patients aged 30–40 (33% to 24%)(Supplementary Fig. 1a). The proportion of patients who were female increased slightly from 47% to 49% (*p*=.01) (Supplementary Fig. 1b).

**Fig. 1:**
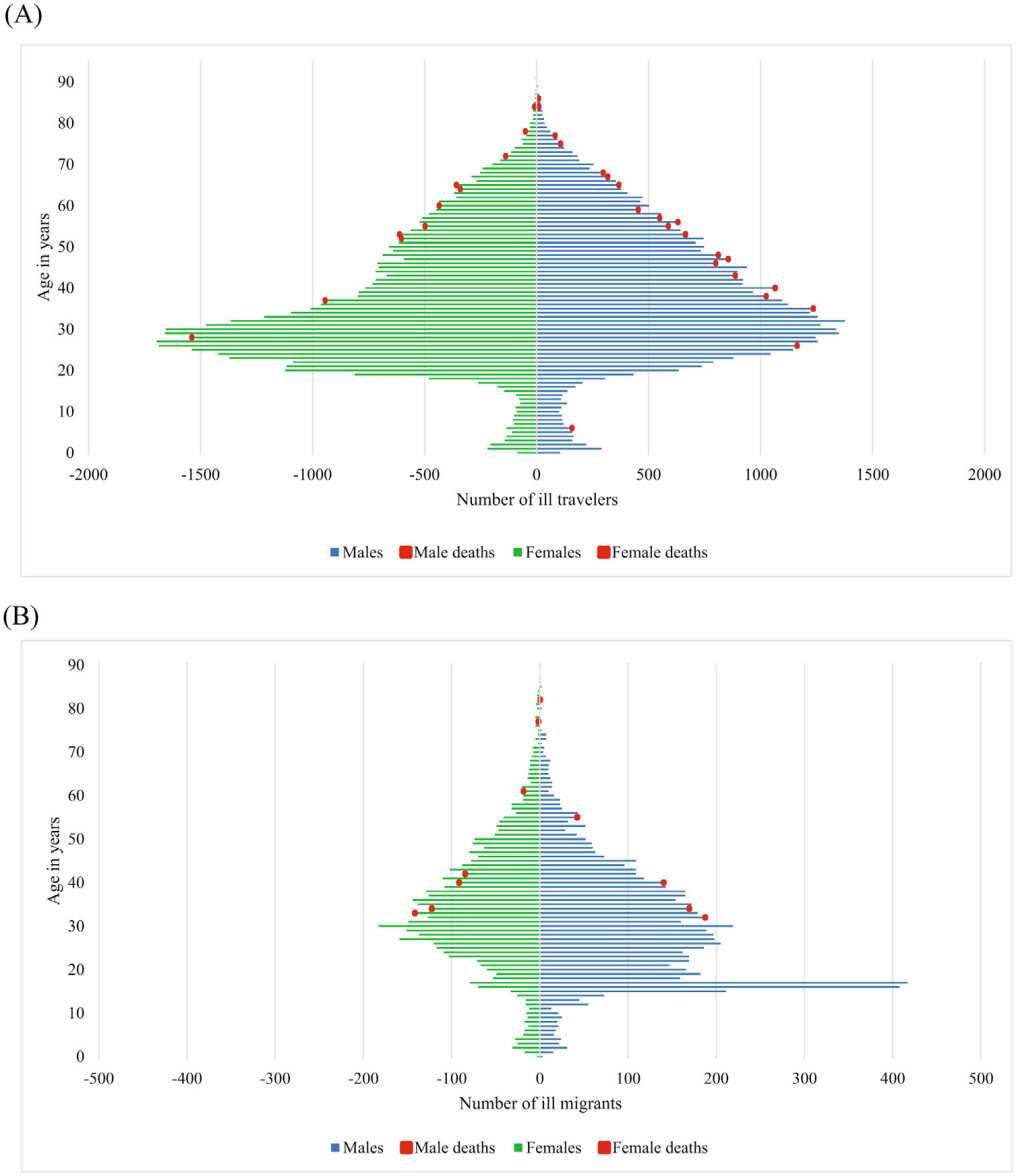
(A) Age and sex for ill travelers seen after or during travel *n* = 92,061 patients with age and sex known, 46,012 males and 46,049 females, 34 deaths. (B) Age and sex for ill travelers with migration travel only. *N* = 11,201 patients with age and sex known, 6737 males and 4473 females, 11 deaths.

**Fig. 2:**
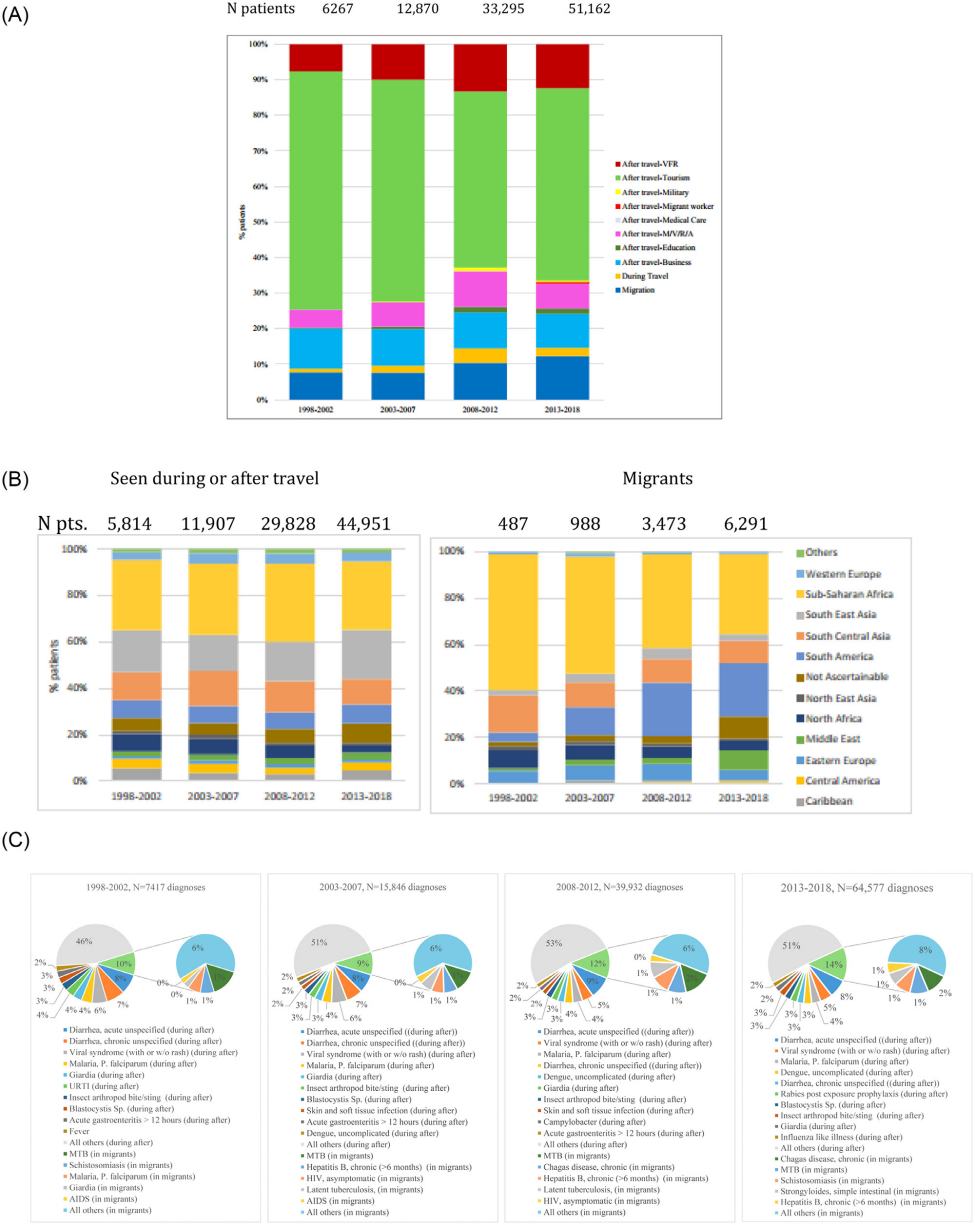
(A) Travel reason and clinical setting by year of clinic visit; *n* = 103,739 patients. (B) Region of exposure by year of clinic visit; *n* = 103,739 patients. (C) Top diagnoses in all patients by clinical setting by year of clinic visit; *n* = 127,772 diagnoses. Multiple entries per patient possible.

There is a significant association between reason for travel and year of clinic visit (*p*<.0001). Analysing in 5-year increments, types of patients seen are becoming more diverse. Whilst business travel-associated illness remained stable, the association was driven by a decrease in the proportion of patients travelling for tourism, and correspondingly increasing proportions mainly of patients who were migrants or visited friends and relatives (VFR; 11% (11,239/103,739; 10.8%) (Figs. 1a,b; 2a; Supplementary Fig. 1c).

**Fig. 3:**
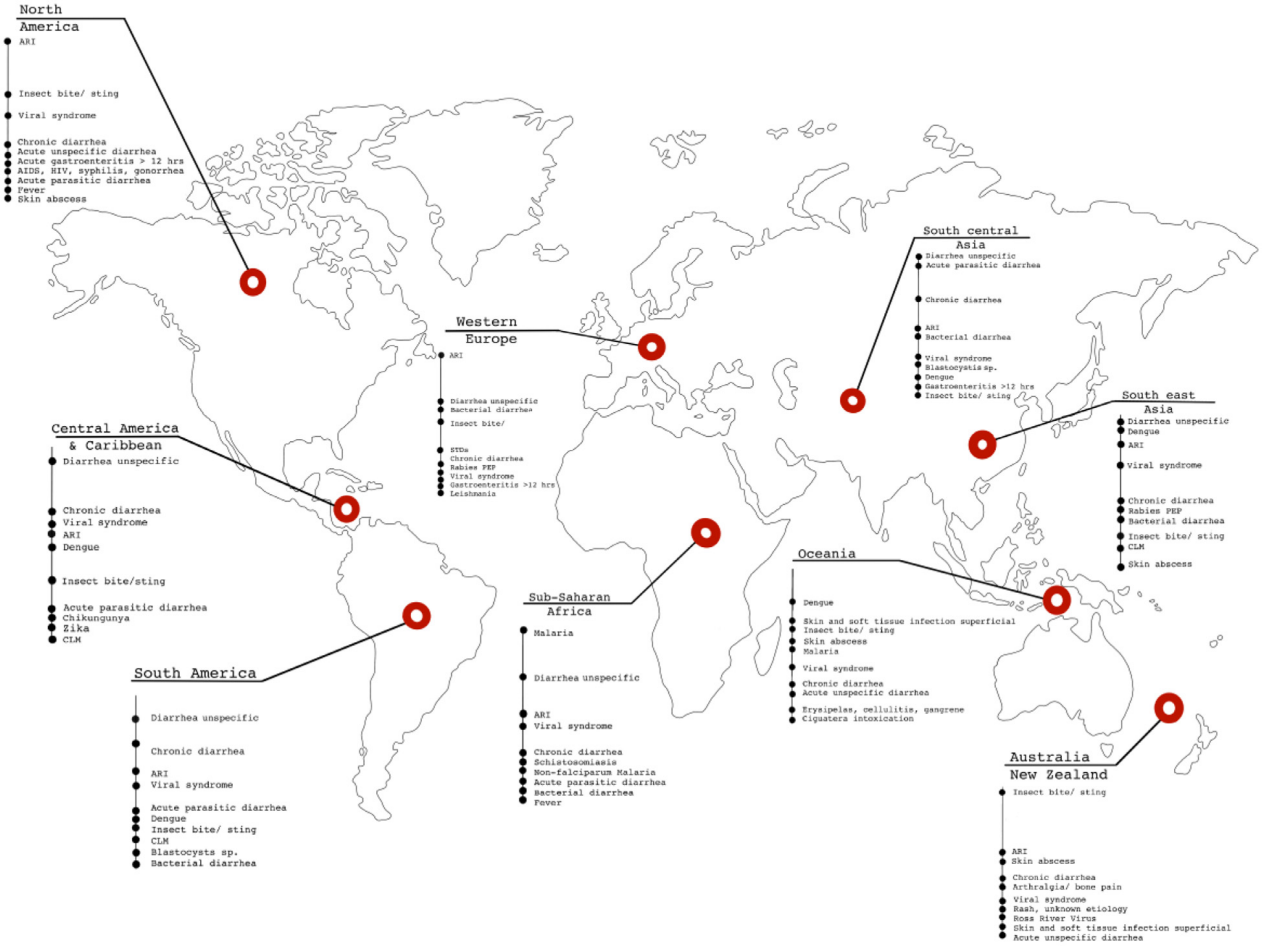
Top 10 diagnoses by region of acquisition in travelers seen after travel.

Regarding top countries of exposure, small changes over time were seen, including for example decreases in the proportion visiting the Dominican Republic, and increases in the proportion exposed in Cameroon and Tanzania. There was a consistent decline in the proportion of ill travellers visiting North Africa (Egypt in particular) from Europe over the past two decades. Over all patient groups, the predominant region of exposure remained sub-Saharan Africa (Fig. 2b), followed by Southeast Asia and the Indian subcontinent (Supplementary Fig. 1d). Fig. 2c presents the top 12 diagnoses in all patients by clinical setting, and by year of clinic visit (127,772 diagnoses). Fig. 3 depicts the top diagnoses per geographical region (see Supplementary Table 2 for further details). Among 111,784 diagnoses in the 92,500 patients seen during and after travel, acute diarrhea (*n* = 10,395; 9.3%), viral syndromes with or without rash (*n* = 6507; 5.8%) and *Plasmodium* falciparum malaria (*n* = 4897, 4.4%) were the top three diagnoses. The top three of 15,988 diagnoses in 11,239 migrants, resulting mainly from screening activities rather than constituting acute diagnoses, were chronic Chagas (*Trypanosoma cruzi*) disease (*n* = 1606; 10.1%), chronic hepatitis C (*n* = 1156; 7.2%), and latent tuberculosis (*n* = 1074; 6.7%). Table 1 provides an overview on selected dignoses encountered over time;

**Table 1:** Selected diagnoses reported between 1998 and 2018 (% of 103,739 patients).

Diagnosis	1998–2002	2003–2007	2008–2012	2013–2018	Somers’ D
Malaria	526 (8.4%)	872 (6.8%)	2340 (7.0%)	3457 (6.8%)	
Dengue	104 (1.7%)	308 (2.4%)	1133 (3.4%)	2176 (4.2%)	0.013*
Chikungunya	0	50 (0.4%)	86 (0.3%)	608 (1.2%)	0.007*
Zika, vector-associated	0	0	0	414 (0.8%)	0.007*
Zika, not vector-associated	0	0	0	6	
Ross River	0	0	5	14	
Yellow fever	0	0	0	5	
Japanese encephalitis	0	0	1	4	
Tick-borne encephalitis	0	5	4	4	
West Nile	0	1	3	3	
Rift Valley Fever	0	0	2	1	
Barmah Forest	0	0	0	1	
Murray Valley encephalitis	0	0	0	1	
*Other arbovirus infections*⁎⁎	0	3	4	6	
All arbovirus diagnoses	104 (1.7%)	364 (2.8%)	1236 (3.7%)	3191 (6.2%)	0.026*
Viral haemorrhagic fever	1	1	12	30	0.0003*
Animal exposure leading to rabies vaccination	41(0.7%)	222(1.7%)	602 (1.8%)	1823 (3.6%)	0.016*
Influenza A and B	0	9 (0.1%)	158 (0.5%)	469 (0.9%)	0.006*
Influenza-like-illness	18 (0.3%)	92 (0.7%)	551 (1.7%)	1295 (2.5%)	0.12*
Acute hepatitis A or B	58 (0.9%)	59 (0.5%)	123 (0.4%)	103 (0.2%)	−0.003*
Measles	4 (0.1%)	3 (0.0%)	21 (0.1%)	17(0.0%)	
Viral syndrome with or without rash	464 (7.4%)	1023 (7.9%	1851 (5.6%)	3225 (6.29%)	
Upper respiratory tract infection	265 (4.2%)	301 (2.3%)	660 (2.0%)	1008 (2.0%)	−0.005*
Total patients	6301	12,895	33,301	51,242	

+Somers’ D only calculated when *N* > 40 and proportions are monotonically increasing or decreasing over time.

⁎ =significant at 95% level.

⁎⁎ Others include sandfly fever/pappataci fever, sindbis fever, unspecified alphavirus, flavivirus and phlebovirus, unspecified arbovirus, 52 patients have 2 arboviral infections.

### 3.2 Deaths

The overall death proportion was low (0.04%), with 45 deaths recorded in the 20-year observation period (Fig. 1A,B). One six-year-old from the UK died from viral encephalitis (unconfirmed tick-borne encephalitis) with exposure in Germany. All other deaths occurred beyond the age of 30 years. Sixty-two percent of deaths occurred in men, who constituted only 51.1% of all ill patients reported. Among non-migrants, men were more likely to die than women (23/46,199 = 0.05% vs. 11/46,196 = 0.02%, *p*=.04), although among migrants, more women died (6/4481 = 0.13% vs. 5/6748 = 0.07%, *p*=.135). Proportionally, 2.7 times more migrants died compared to non-migrants (11/11,229 = 0.1%, and 34/92,395 = 0.04%, respectively). Overall, malaria patients had a 2.5:1 risk ratio of dying from their disease compared to patients with all other diagnoses (7/7195 = 0.1% vs 38/96,544 = 0.04%). See Supplementary Table 3a,b for details of frequencies of causes of death in travellers’ cohorts and causes of death in this cohort.

### 3.3 Malaria

**Fig. 4.**
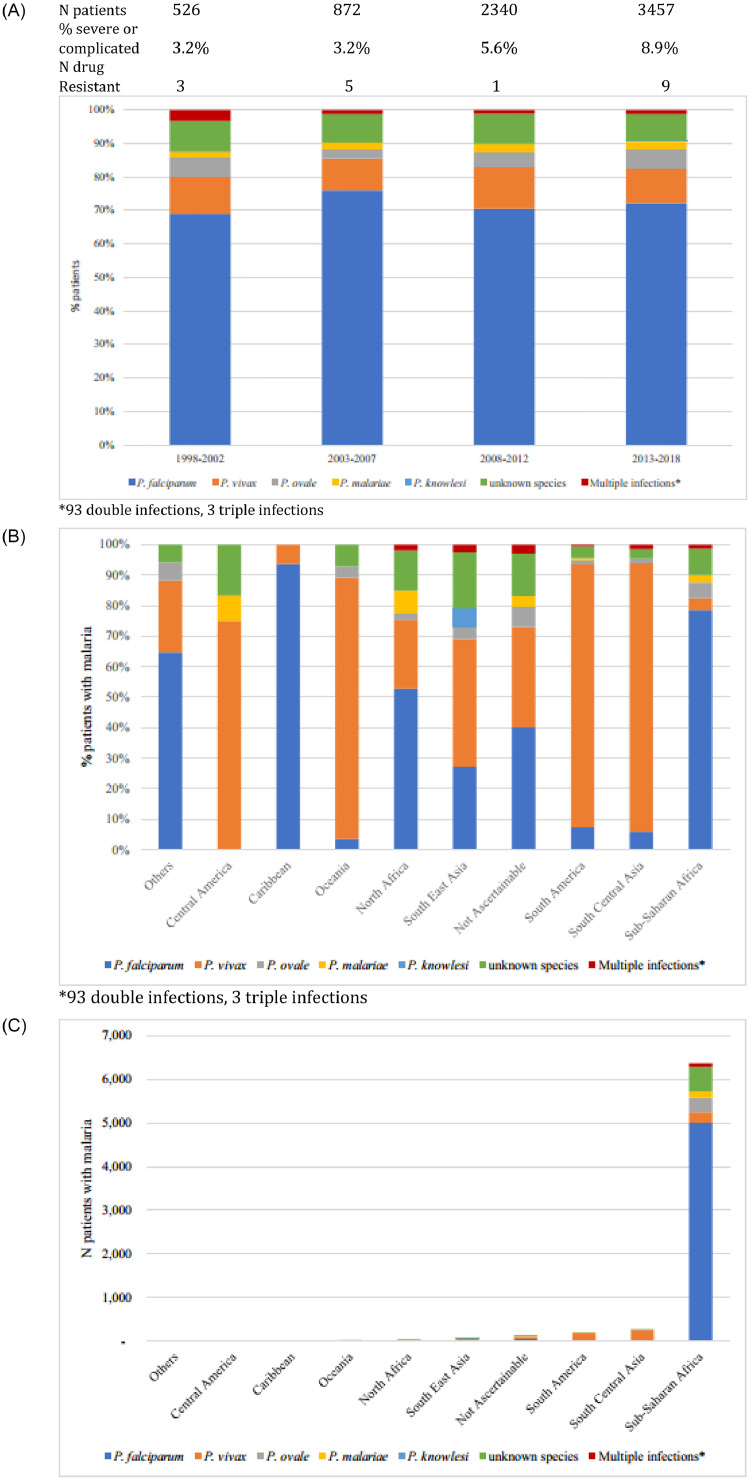
(A) Malaria species by 5 years, shown by% of malaria diagnoses. (B) Origin of malaria as *Plasmodium* species per region of acquisition, shown by% of malaria diagnoses per region. (C) Origin of malaria as *Plasmodium* species per region of acquisition, shown as numbers of patients.

Malaria continues to be the number two diagnosis for patients seen during and after travel. Of all patients (travelers and migrants combined), 7195 (6.9%) had malaria. Of those, 6370 (88.5%) were from sub-Saharan Africa, of which 5082 (79.8%) had *P. falciparum* malaria. Overall, *P. falciparum* malaria ranked third in this 20-year analysis with 5254 cases (5.1% of all patients) (Fig. 2c, 4a-c).

3407 of 7195 (47.3%) of the malaria cases were in VFRs. The seasonality of EuroTravNet malaria diagnoses was stable, with an annual peak from June to October, correlating with school holidays. Among 11,239 migrants, 337 (3.0%) had *P. falciparum* malaria and 204 (1.8%) *P. vivax* malaria. *P. knowles*i infections were reported in five tourists and business travelers who had visited Malaysia (three) and Thailand (two). A recent peak of *P. vivax* in 2014–2015 with more than 100 cases annually in refugees is now back to pre-migration levels of 50–60 cases per year [9,11].

### 3.4 Arboviral diseases and viral haemorrhagic fevers

There were significant increases in dengue diagnoses, in chikungunya diagnoses, in Zika diagnoses and in other arboviral diseases. In addition, the range of detected arboviral diseases increased among all ill patients (Table 1; Fig. 2c). More recently, increases in imported yellow fever and West Nile fever cases were observed, and as well a surge of Zika cases during the recent large outbreaks in the Americas and globally [12,13].

According to case definition, there were 44 cases of viral haemorrhagic fever (VHF) seen in our cohort. The majority (*n* = 29; 66.1%) were severe dengue infections. Of note, with the exception of one patient with yellow fever reported by the network [14] and one with dengue haemorrhagic fever, all patients survived. Additional VHF patients included five with yellow fever, three with Ebola virus disease, three with Rift Valley fever, two with hantavirus, one with Crimean-Congo hemorrhagic fever (CCHF) and one with Lassa fever. There was a small significant increase in VHF cases over the study period with five VHF cases reported in the first quarter of 2018 alone (Supplementary Fig. 2).

### 3.5 Rabies

Animal exposures leading to rabies post-exposure prophylaxis (RPEP) significantly increased over the time period and accounted for 2.6% (2688/103,739) of all patients seen over the study period. Tourists accounted for the highest proportion (80.9%) of RPEP, followed by VFR travelers (11.3%). Overall, dogs were responsible for 46.1% of exposures followed by monkeys (22.8%), cats (12.7%) and bats (2.0%). Most travellers were exposed in Asia (53.8%), mainly southeast Asia (42.1%), followed by North Africa (12.4%), Latin America (8.8%), Middle East (7.5%) and sub-Saharan Africa (6.9%). In southeast Asia, monkeys were responsible for 43.3% of exposures.

### 3.6 Rare diagnoses

Unusual diagnoses are reported as well (Supplementary Table 4). There were significant increases in four unusual diagnoses, such as hepatic echinococcosis; leptospirosis; legionnaires disease and Shiga toxin-producing *E. coli.*

### 3.7 Vaccine preventable diseases (VPD)

Seasonal influenza, the most commonly diagnosed VPD, accounted for 0.6% (636/103,739) of all patients and increased significantly over the study period. Although not necessarily a VPD, proportions of patients with diagnoses of influenza-like-illness also increased significantly (Fig. 2C; Supplementary Fig. 3). There was a surge in 2009 in the number of patients reported with the novel H1N1 influenza strain (from 6 previously to 109).

The next most-common VPDs were hepatitis A (219 patients) and hepatitis B (125 patients) which decreased as a percentage of ill patients over the study period. Other common VPDs were varicella (78 patients), pertussis (58), measles (45), mumps (25), rubella (14), TBE (13), diphtheria (8), yellow fever (5), and Japanese encephalitis (5). Most VPDs were seen during or post-travel, although >20% of varicella patients (16/78), hepatitis B patients (33/125) and mumps patients (6/25) were migrants. The greatest proportion of VPDs was acquired in North America (primarily influenza), during air travel (primarily varicella) and in the Middle East (primarily influenza, especially from Saudi Arabia). The proportion of patients with a VPD who had sought pre-travel advice was 0.8% while it was 1.7% and 1.4% in those who had not, or whose status was not known, respectively. The highest proportion of VPDs was seen in elderly travellers aged 70 and older (49/2570; 1.9%), and young travellers aged 9 years and younger (55/2952; 1.9%). Looking specifically at measles, which has been undergoing a global resurgence, revealed the following regions of acquisition for the 45 patients with measles: 16 from southeast Asia, 9 from sub-Saharan Africa, 7 from western Europe, 5 from South Central Asia and 8 from various other regions. Some of the most common countries of exposure included Thailand (8), Indonesia and France (4 each), and India (3). There was no increase over time in proportions of patients with measles.

## 4 Discussion

Analyses of surveillance data constitute a knowledge base for travel medicine practice.

The strengths of the data include the large number of clinician-verified patient records (>100,000) on travel-associated illness, itinerary data, the 20-year data collection period allowing analysis over time, and the geographic representation of the European network sites. These factors ensure a realistic representation of travel-related illness in people seeking medical care in Europe. Also the size of the network improves the ability to use travelers as sentinels for disease outbreaks.

Over time, the number of EuroTravNet sites and participating countries has grown and some sites have left the network; however, the network also has reflected changes in travel patterns. The origin of migrants did change over time, due mainly to the additional Spanish sites seeing migrants from South America, and changes in the origin of major waves of migrants arriving in Europe. Migrants from the Horn of Africa, mainly from Eritrea, led to a surge of *P. vivax* malaria reporting in recent years, in contrast to a traditional dominance of *P. falciparum* in West African migrants presenting in Europe [15].

Travel medicine needs to continue to adapt to global health challenges and changing disease epidemiology, in terms of, simplified, ‘who travels where to, and how’. It needs to account for (1) an increasing diversity and expansion of destinations; (2) a shift from ‘high income to middle-and low income country travel to a more diverse mixture of global movement; (3) the fact that the term ‘traveller’ should be interpreted broader and include migrants; (4) a finer granularity of creasingly accurate final diagnoses due to improved diagnostic capacities and capabilities; (5) a decrease in geopgraphic pixel size; (6) all aspects of post ’travel’ (in the broad sense of the word) care for vulnerable, immune-compromised, migrant and VRF individuals and populations; and (7), often discrete but meaningful changes in the composition of the mixture of travellers’ types, their age pattern, and the spectrum and distribution of exposure countries.

With the massive volume growth of international travel, there is an ever-increasing number of travellers in terms of their origin as well as a diversification and expansion of destinations. In 1950, 97% of all tourists visited 15 top destinations whereas in 2015, only 54% of tourist arrivals were registered at these top destinations [3], with people visiting a wider range of remote destinations.

Travel medicine focused almost exclusively on travel preparation and disease prevention. Now, the post-travel care component has emerged to share center-stage. Travel medicine previously focused on the traveler from high-income to middle- and low-income countries, whereas now, the care of travellers is much more multi-faceted. The term ‘traveller’ encompasses all cross-border movements globally, and includes migrants and VFRs as increasingly important groups; with the next step being to decrease ‘pixel size’ by acknowledging that in-country and intra-regional travel needs to be added to our definition of what constitutes a traveller, particularly if different ecosystems are spanned by one country's borders [16]. Reporting diligence, moving from analog to digital; bandwidth and accuracy of diagnoses as well as network sizes have expanded range and granularity of sentinel surveillance dramatically. Challenges, however, arise from the changing profile of travel-related illness, with rapid public health responses needed in case of highly-infectious, imported diseases such as filoviruses, Middle East respiratory syndrome coronavirus (MERS-CoV), and monkeypox. A major trend is the emergence of greater numbers of ill travellers with arboviral diseases (possibly owed to a combination of more travellers being exposed, hence greater concern and greater numbers diagnosed because of improvements in diagnostics), with increases in dengue and chikungunya and the risk of introducing these infections into areas of southern Europe with susceptible vector populations and the emergence of not previously recorded infections such as Ross River virus disease, Japanese encephalitis, and Zika. This trend dictates that arthropod-bite prevention must become a central theme in travel medicine [17]. Obviously, the epidemics in Latin America of chikungunya in 2013, and Zika in 2015/2016, and recent outbreaks of yellow fever in Brazil and Nigeria have led to increased diagnostic testing, which is a reflection of the growing appreciation of the clinical importance of these infections, which may have been grossly under-recognised in the past, and which may have brought unnecessary suffering upon patients, uncertain about their unexplained complaints. Another challenge is the emergence of influenza, demanding improvements in diagnostics and case definitions for influenza-like-illness.

Recognition of VPD not classically confined to ‘the tropics’ must become firmly integrated into routine pre-travel counselling. The pre-travel consultation presents an ideal opportunity to catch up on routine or missed vaccinations. The increasing risk of acquiring diseases in high-income countries is exemplified by measles [18,19] due to increasing vaccine hesitancy [20], and should capitalize on trends towards inoculation schemes that require less antigen, such as the intradermal route of application [21]. The recent changes in rabies [22] and yellow fever vaccination [23] regimens are prominent examples.

The occurrence of documented transmission of rabies from non-human primates (NHPs) to humans, although rare, implies that RPEP is indicated in patients injured by NHPs in rabies-enzootic countries [24], [25], [26], [27]. Although there has been an increase in bites resulting in RPEP over the 20 years, it is likely that a significant proportion of tourists receiving RPEP following injuries caused by NHP in Asia are actually bitten by uninfected animals. Measures aiming at reducing contacts with NHP should be promoted among travellers. In addition, our analysis did not suggest an acute increase in the proportion of ill travellers with measles, although the database only includes patients until early 2018. However, a recent GeoSentinel analysis of all patients with measles reported since 2016 [28] revealed an increase in confirmed and probable measles cases between 2016 and 2018, concurrent with the global resurgence of this disease [29]. There is thus a clear need for pre-travel health providers to review measles vaccination status with travellers and make sure that they have been fully immunised.

There is also a need to address inadequate post-travel care for vulnerable and hard-to-reach populations such as migrants and VFR-travellers. Their health care teams should incorporate travel medicine specialists, and recognise problems of public health importance such as the importation and establishment of autochthonous ‘tropical diseases’ in previously unafflicted areas (such as CCHF in Spain, chikungunya and other arthropod-borne viral diseases, or malaria in Southern European countries), and the exportation of VPDs by migrants fleeing economic instability and war [9,30]. Finally, pre-travel care for immuno-compromised patients is an important evolving field within travel medicine; with the advent of powerful, targeted immunosuppressive medication, this patient group has become increasingly mobile and requires targeted travel medicine. Research on adapted vaccination strategies in this patient group is urgently required [31,32].

With any analysis and interpretation of sentinel surveillance data, an appreciation of the limitations of this methodological approach is paramount. Denominator data are missing and the data presented here focus primarily on more severe infectious diseases and provide a synopsis of what travel medicine clinicians in specialised clinics typically diagnose. These data do not reflect precisely the full range of infectious illnesses contracted by travellers as all self-resolving conditions and those that are diagnosed by a general practitioner are not captured. A limitation of our study is that mortality data do not allow calculation of mortality risk for travelers, as our data do not capture the vast majority of deaths that are of non-infectious causes or due to accidents and injuries [33]. Furthermore, our data do not capture deaths of European travelers occurring during travel outside Europe.

Another limitation is the changing number and profile of sites in EuroTravNet over time and therefore, analyses over time must be interpreted with some caution. In addition, diagnostic capacity has changed during the last 20 years and the sites’ capacity to make specific etiological diagnoses has improved.

From a niche sub-specialty, travel medicine has emerged, and continues to evolve.

Over time, there has been increased migrant and VFR travel. Arthropod bite prevention strategies must be improved, given the global increase in arthropod-borne infections, and the risk of their potential introduction into Europe in view of climate change and widespread suitable vector prevalence.

Travel medicine is a multidisciplinary field that needs to recognise emerging and changing infectious disease trends and to inform the public, and tailor pre-travel advice to individual needs accordingly. It also needs to broaden its remit to address the health care needs of diverse populations, including migrants, medical tourists, and occupational groups who are at risk of travel-related illness. This speciality must also be alert to the potential for global travellers to disseminate infection and to introduce pathogens to previously unaffected areas.

## Data presentation

Part of these data was presented during CISTM16, June 2019, Washington, DC, USA.

## Author contributions

MPG, LW, AG, DH, PG and PS analysed the data and drafted the manuscript. All authors contributed to data collection, interpretation and writing, and approved the final version of the manuscript.

## EuroTravNet collaborators

Kees Stijnis, Michèle van Vugt (Center of Tropical Medicine and Travel Medicine, Amsterdam University Medical Centers, location AMC, Amsterdam, The Netherlands); Camilla Rothe, Frank von Sonnenburg (Department of Infectious Diseases and Tropical Medicine (DITM), Ludwig-Maximilians University of Munich, Munich, Germany); Christof Vinnemeier, Michael Ramharter (Division of Tropical Medicine, I. Department of Medicine, University Medical Center Hamburg-Eppendorf & Department of Tropical Medicine, Bernhard Nocht Institute for Tropical Medicine, Hamburg, Germany); Gabriela Equihua Martinez, Maximilian Gertler (Charité-Universitätsmedizin Berlin, corporate member of Freie Universität Berlin, Humboldt-Universität Berlin & Berlin Institute of Health, Institute of Tropical Medicine and International Health, Berlin, Germany); Gilles Eperon (Division of Tropical and Humanitarian Medicine, Geneva University Hospitals, Geneva, Switzerland); Johan Ursing, Hedvig Glans (Department of Infectious Diseases, Karolinska University Hospital, Stockholm, Sweden & Unit of Infectious Diseases, Department of Medicine, Huddinge, Karolinska Institutet, Stockholm, Sweden); Arnhild Hægeland, Mona Joof (Department of Infectious Diseases, Oslo University Hospital, Ullevål, Oslo, Norway); Corine Stuij (Department of Microbiology and Infectious Diseases, University Hospital Erasmus MC, Rotterdam, The Netherlands); Silvia Odolini, M.D.; Lina Tomasoni (University of Brescia & ASST Spedali Civili de Brescia, Brescia, Italy); Sandra Chamorro (National Referral Unit for Tropical Diseases, Infectious Diseases Department, Ramón y Cajal University Hospital Madrid, Madrid, Spain); Ted Lankester (InterHealth Worldwide, Newington Causeway, London, United Kingdom); Fernando Salvador, Nuria Serre-Delcor, (Department of Infectious Diseases, Vall d'Hebron University Hospital, PROSICS Barcelona, Barcelona, Spain); Cécile Ficko (Infectious and Tropical Diseases Department, HIA Bégin, Saint-Mandé, France); Elena Trigo (National Referral Unit for Imported Tropical Diseases, Department of Internal Medicine, Hospital Universitario La Paz-Carlos III, IdiPAZ, Madrid, Spain); Benjamin Warne, (Department of Medicine, University of Cambridge, Cambridge, United Kingdom); Sanne Jespersen, Christian Weijse (Department of Infectious Diseases, Aarhus University Hospital, Aarhus, Denmark); Alexandre Duvignaud, Thierry Pistone (Unit of Tropical Medicine and Clinical International Health, Department of Infectious and Tropical Diseases, University Hospital Center of Bordeaux & INSERM 1219, University of Bordeaux, Bordeaux, France); Nick J. Beeching, Mike B.J. Beadsworth (Liverpool School of Tropical Medicine and Tropical Infectious Disease Unit, Royal Liverpool University Hospital, Liverpool, United Kingdom); Paola Rodari, Lucia Moro (Department of Infectious/Tropical Diseases and Microbiology, IRCCS Sacro Cuore Don Calabria Hospital, Negrar, Verona, Italy); Corneliu P. Popescu, Mihaela F. Zaharia (Dr Victor Babes Clinical Hospital of Infectious and Tropical Diseases, Bucharest, Romania); Emilie Javelle, Philippe Parola (University Hospital Institute Méditerranée Infection, Aix-Marseille Univ, Marseille, France); Rainer Weber (Zürich University Hospital); Sabine Schmid-Stoll (University of Zürich Centre for Travel Medicine, WHO Collaborating Centre for Travellers' Health, Department of Public Health, Institute for Epidemiology, Biostatistics and Prevention, Zürich, Switzerland).

## Data sharing statement

Access to all original data can be requested from the authors.

## Declaration of Competing Interest

None of the authors has a conflict of interest to declare in relation to the manuscript.

## Appendix B Supplementary materials

Image, application 1
