# Supplementary material for: Travel-related infections presenting in Europe: A 20-year analysis of EuroTravNet surveillance data
Source: Lancet Reg Health Eur. 2020 Nov 12;1:100001. doi: 10.1016/j.lanepe.2020.100001 (PMC8454853; doi:10.1016/j.lanepe.2020.100001)
Supplement: Supplementary file 1 [file mmc1.docx]

**Supplementary Tables Legends**

**Supplementary Table 1.** Patient types.

**Supplementary Table 2.** 20 top diagnoses per region of exposure.

**Supplementary Table 3a.** Death records in various GeoSentinel/EuroTravNet sentinel surveillance (sub)cohorts. Note that there is a partial cohort overlap between the various analyses, depending on reporting period, (sub)cohort and analytical approach chosen.

**Supplementary Table 3b.** Causes of death in our cohort described here.

**Supplementary Table 4.** Rare diagnoses.

**Supplementary Figures Legend**

**Supplementary Figure 1a-d.** Group characteristics changes over time by year group.

**Supplementary Figure 1a.** Age group by year group.

**Supplementary Figure 1b.** Sex by year group.

**Supplementary Figure 1c.** Changes in traveller groups over time.

**Supplementary Figure 1d.**

**Supplementary Figure 2.** Viral haemorrhagic fevers.

**Supplementary Figure 3.** Vaccine preventable diseases.

**Supplementary Tables**

**Supplementary Table 1.** Patient types.

| **Patient Type** | Migration travel only | % | Seen after travel* | % | Seen during travel* | % | Total |
| --- | --- | --- | --- | --- | --- | --- | --- |
| Inpatient | 2,696 | 24.0 | 11,310 | 12.6 | 662 | 23.0 | 14,668 |
| Outpatient | 6,557 | 58.3 | 78,134 | 87.2 | 2,213 | 76.8 | 86,904 |
| Missing | 1,986 | 17.7 | 176 | 0.2 | 5 | 0.2 | 2,167 |
| Total | 11,239 | 100.0 | 89,620 | 100.0 | 2,880 | 100.0 | 103,739 |

*Non-migrant travelers only

**Supplementary Table 2.** Top 20 diagnoses per region of exposure.

| **North East Asia N=1433** | **Freq** | **%** | **South Central Asia N=14028** | **Freq** | **%** | **South East Asia N=21395** | **Freq** | **%** | **Oceania N=470** | **Freq** | **%** |
| --- | --- | --- | --- | --- | --- | --- | --- | --- | --- | --- | --- |
| RESPIRATORY INFECTION, ACUTE | 189 | 13.2 | DIARRHEA, ACUTE UNSPECIFIED | 2149 | 15.3 | DIARRHEA, ACUTE UNSPECIFIED | 2289 | 10.7 | SKIN & SOFT TISSUE INFECTIONS | 72 | 15.3 |
| DIARRHEA, ACUTE UNSPECIFIED | 187 | 13.0 | DIARRHEA ACUTE parasitic | 1742 | 12.4 | DENGUE, UNCOMPLICATED | 1797 | 8.4 | DENGUE, UNCOMPLICATED | 37 | 7.9 |
| DIARRHEA, CHRONIC | 133 | 9.3 | DIARRHEA, CHRONIC | 1192 | 8.5 | RESPIRATORY INFECTION, ACUTE | 1698 | 7.9 | INSECT OR OTHER ARTHROPOD BITE/STING (WITH OR WITHOUT SUPRAINFECTION) | 28 | 6.0 |
| VIRAL SYNDROME (WITH/WITHOUT RASH) | 93 | 6.5 | RESPIRATORY INFECTION, ACUTE | 899 | 6.4 | VIRAL SYNDROME (WITH/WITHOUT RASH) | 1524 | 7.1 | MALARIA (ALL SPECIES) | 28 | 6.0 |
| RABIES PEP (post exposure prophylaxis) | 54 | 3.8 | DIARRHEA ACUTE BACTERIAL | 877 | 6.3 | SKIN & SOFT TISSUE INFECTIONS | 1178 | 5.5 | VIRAL SYNDROME (WITH/WITHOUT RASH) | 25 | 5.3 |
| ANXIETY/FATIGUE | 48 | 3.3 | VIRAL SYNDROME (WITH/WITHOUT RASH) | 623 | 4.4 | DIARRHEA, CHRONIC | 1154 | 5.4 | DIARRHEA, ACUTE UNSPECIFIED | 23 | 4.9 |
| DIARRHEA ACUTE BACTERIAL | 44 | 3.1 | BLASTOCYSTIS Sp. | 589 | 4.2 | RABIES PEP (post exposure prophylaxis) | 1130 | 5.3 | RESPIRATORY INFECTION, ACUTE | 20 | 4.3 |
| DIARRHEA ACUTE parasitic | 43 | 3.0 | SKIN & SOFT TISSUE INFECTIONS | 559 | 4.0 | DIARRHEA ACUTE BACTERIAL | 1085 | 5.1 | DIARRHEA, CHRONIC | 17 | 3.6 |
| BITE, ANIMAL | 40 | 2.8 | DENGUE, UNCOMPLICATED | 482 | 3.4 | BITE, ANIMAL | 1064 | 5.0 | CHIKUNGUNYA VIRUS INFECTION | 11 | 2.3 |
| BLASTOCYSTIS Sp. | 37 | 2.6 | INSECT OR OTHER ARTHROPOD BITE/STING (WITH OR WITHOUT SUPRAINFECTION) | 319 | 2.3 | INSECT OR OTHER ARTHROPOD BITE/STING (WITH OR WITHOUT SUPRAINFECTION) | 901 | 4.2 | CIGUATERA INTOXICATION | 10 | 2.1 |
| INSECT OR OTHER ARTHROPOD BITE/STING (WITH OR WITHOUT SUPRAINFECTION) | 33 | 2.3 | RABIES PEP (post exposure prophylaxis) | 258 | 1.8 | CUTANEOUS LARVA MIGRANS, HOOKWORM-RELATED | 797 | 3.7 | DIARRHEA ACUTE parasitic | 10 | 2.1 |
| SKIN & SOFT TISSUE INFECTIONS | 30 | 2.1 | BITE, ANIMAL | 235 | 1.7 | DIARRHEA ACUTE parasitic | 594 | 2.8 | BLASTOCYSTIS Sp. | 9 | 1.9 |
| AIDS, HIV, SYPHILIS, GONORRHEA | 19 | 1.3 | MALARIA (ALL SPECIES) | 213 | 1.5 | BLASTOCYSTIS Sp. | 389 | 1.8 | ANXIETY/FATIGUE | 8 | 1.7 |
| ABDOMINAL PAIN, UNSPECIFIED ETIOLOGY | 18 | 1.3 | ANXIETY/FATIGUE | 184 | 1.3 | AIDS, HIV, SYPHILIS, GONORRHEA | 318 | 1.5 | DIARRHEA ACUTE BACTERIAL | 8 | 1.7 |
| HEPATITIS (VIRAL- ACUTE) | 18 | 1.3 | FEBRILE ILLNESS, UNSPECIFIED (< 3 WEEKS) | 180 | 1.3 | FEBRILE ILLNESS, UNSPECIFIED (< 3 WEEKS) | 295 | 1.4 | MARINE ENVENOMATION/FISH STING | 8 | 1.7 |
| URINARY TRACT INF, ACUTE | 17 | 1.2 | URINARY TRACT INF, ACUTE | 152 | 1.1 | RASH, UNKNOWN ETIOLOGY (NON-FEBRILE) | 265 | 1.2 | Animal bite w/o rabies PEP | 7 | 1.5 |
| DENGUE, UNCOMPLICATED | 16 | 1.1 | SALMONELLA TYPHI | 139 | 1.0 | ANXIETY/FATIGUE | 248 | 1.2 | FEBRILE ILLNESS, UNSPECIFIED (< 3 WEEKS) | 7 | 1.5 |
| EPSTEIN-BARR VIRUS | 15 | 1.0 | NON PATHOGENIC PROTOZOA (not Blastocystis) | 134 | 1.0 | URINARY TRACT INF, ACUTE | 217 | 1.0 | RASH, UNKNOWN ETIOLOGY (NON-FEBRILE) | 7 | 1.5 |
| ARTHRALGIA/BONE PAIN | 14 | 1.0 | NEMATODE INFECTIONS, INTESTINAL | 129 | 0.9 | NEMATODE INFECTIONS, INTESTINAL | 197 | 0.9 | ARTHRALGIA/BONE PAIN | 6 | 1.3 |
| ARTHRITIS, NONSEPTIC | 14 | 1.0 | RASH, UNKNOWN ETIOLOGY (NON-FEBRILE) | 116 | 0.8 | FUNGAL INFECTION (SUPERFICIAL/SUBCUTANEOUS/CUTANEOUS MYCOSIS) | 166 | 0.8 | NEMATODE INFECTIONS, INTESTINAL | 6 | 1.3 |

| **South America N=8511** | **Freq** | **%** | **Central America N=3568** | **Freq** | **%** | **Caribbean N=4378** | **Freq** | **%** | **North America N=793** | **Freq.** | **%** |
| --- | --- | --- | --- | --- | --- | --- | --- | --- | --- | --- | --- |
| DIARRHEA, ACUTE UNSPECIFIED | 866 | 10.18 | DIARRHEA, ACUTE UNSPECIFIED | 494 | 13.85 | DIARRHEA, ACUTE UNSPECIFIED | 499 | 11.40 | RESPIRATORY INFECTION, ACUTE | 186 | 23.46 |
| DIARRHEA, CHRONIC | 623 | 7.32 | DIARRHEA, CHRONIC | 346 | 9.70 | VIRAL SYNDROME (WITH/WITHOUT RASH) | 376 | 8.59 | INSECT OR OTHER ARTHROPOD BITE/STING (WITH OR WITHOUT SUPRAINFECTION) | 68 | 8.58 |
| RESPIRATORY INFECTION, ACUTE | 530 | 6.23 | RESPIRATORY INFECTION, ACUTE | 269 | 7.54 | DENGUE, UNCOMPLICATED | 347 | 7.93 | DIARRHEA, ACUTE UNSPECIFIED | 47 | 5.93 |
| VIRAL SYNDROME (WITH/WITHOUT RASH) | 474 | 5.57 | VIRAL SYNDROME (WITH/WITHOUT RASH) | 211 | 5.91 | RESPIRATORY INFECTION, ACUTE | 288 | 6.58 | VIRAL SYNDROME (WITH/WITHOUT RASH) | 42 | 5.30 |
| DIARRHEA ACUTE parasitic | 393 | 4.62 | INSECT OR OTHER ARTHROPOD BITE/STING (WITH OR WITHOUT SUPRAINFECTION) | 193 | 5.41 | DIARRHEA, CHRONIC | 277 | 6.33 | SKIN & SOFT TISSUE INFECTIONS | 33 | 4.16 |
| DENGUE, UNCOMPLICATED | 375 | 4.41 | DENGUE, UNCOMPLICATED | 176 | 4.93 | CHIKUNGUNYA VIRUS INFECTION | 247 | 5.64 | DIARRHEA, CHRONIC | 27 | 3.40 |
| INSECT OR OTHER ARTHROPOD BITE/STING (WITH OR WITHOUT SUPRAINFECTION) | 349 | 4.10 | DIARRHEA ACUTE parasitic | 167 | 4.68 | INSECT OR OTHER ARTHROPOD BITE/STING (WITH OR WITHOUT SUPRAINFECTION) | 217 | 4.96 | AIDS, HIV, SYPHILIS, GONORRHEA | 23 | 2.90 |
| SKIN & SOFT TISSUE INFECTIONS | 317 | 3.72 | SKIN & SOFT TISSUE INFECTIONS | 120 | 3.36 | ZIKA (includes screening) | 185 | 4.23 | DIARRHEA ACUTE parasitic | 17 | 2.14 |
| CUTANEOUS LARVA MIGRANS, HOOKWORM-RELATED | 300 | 3.52 | BLASTOCYSTIS Sp. | 112 | 3.14 | SKIN & SOFT TISSUE INFECTIONS | 152 | 3.47 | BITE, ANIMAL | 16 | 2.02 |
| BLASTOCYSTIS Sp. | 260 | 3.05 | CUTANEOUS LARVA MIGRANS, HOOKWORM-RELATED | 104 | 2.91 | CUTANEOUS LARVA MIGRANS, HOOKWORM-RELATED | 149 | 3.40 | RASH, UNKNOWN ETIOLOGY (NON-FEBRILE) | 14 | 1.77 |
| DIARRHEA ACUTE BACTERIAL | 217 | 2.55 | DIARRHEA ACUTE BACTERIAL | 94 | 2.63 | DIARRHEA ACUTE parasitic | 117 | 2.67 | RABIES PEP (post exposure prophylaxis) | 13 | 1.64 |
| NEMATODE INFECTIONS, INTESTINAL | 205 | 2.41 | ZIKA (includes screening) | 71 | 1.99 | DIARRHEA ACUTE BACTERIAL | 110 | 2.51 | ANXIETY/FATIGUE | 12 | 1.51 |
| MALARIA (ALL SPECIES) | 199 | 2.34 | RABIES PEP (post exposure prophylaxis) | 65 | 1.82 | ANXIETY/FATIGUE | 83 | 1.90 | MARINE ENVENOMATION/FISH STING | 12 | 1.51 |
| CHIKUNGUNYA VIRUS INFECTION | 159 | 1.87 | Animal bite w/o rabies PEP | 57 | 1.60 | BLASTOCYSTIS Sp. | 74 | 1.69 | BITE, TICK | 11 | 1.39 |
| CHAGAS DISEASE, CHRONIC | 152 | 1.79 | NEMATODE INFECTIONS, INTESTINAL | 57 | 1.60 | RASH, UNKNOWN ETIOLOGY (NON-FEBRILE) | 65 | 1.48 | FEBRILE ILLNESS, UNSPECIFIED (< 3 WEEKS) | 11 | 1.39 |
| RABIES PEP (post exposure prophylaxis) | 143 | 1.68 | RASH, UNKNOWN ETIOLOGY (NON-FEBRILE) | 54 | 1.51 | FEBRILE ILLNESS, UNSPECIFIED (< 3 WEEKS) | 55 | 1.26 | LYME DISEASE - ACUTE OR EARLY DISEASE (INCLUDING ERYTHEMA CHRONICUM MIGRANS AND OTHER MANIFESTATION | 11 | 1.39 |
| Animal bite w/o rabies PEP | 129 | 1.52 | FEBRILE ILLNESS, UNSPECIFIED (< 3 WEEKS) | 47 | 1.32 | AIDS, HIV, SYPHILIS, GONORRHEA | 53 | 1.21 | URINARY TRACT INF, ACUTE | 11 | 1.39 |
| AIDS, HIV, SYPHILIS, GONORRHEA | 124 | 1.46 | ANXIETY/FATIGUE | 42 | 1.18 | URINARY TRACT INF, ACUTE | 53 | 1.21 | MENINGITIS | 8 | 1.01 |
| MYIASIS | 123 | 1.45 | MYIASIS | 42 | 1.18 | NEMATODE INFECTIONS, INTESTINAL | 42 | 0.96 | RASH, DERMATITIS (incl contact dermatitis) | 8 | 1.01 |
| ZIKA (includes screening) | 122 | 1.43 | LEISHMANIA | 41 | 1.15 | ARTHRALGIA/BONE PAIN | 39 | 0.89 | ARTHRALGIA/BONE PAIN | 7 | 0.88 |

| **Sub-Saharan Africa N=34028** | **Freq** | **%** | **North Africa N=5175** | **Freq** | **%** | **Western Europe N=4392** | **Freq** | **%** | **Eastern Europe N=1609** | **Freq** | **%** |
| --- | --- | --- | --- | --- | --- | --- | --- | --- | --- | --- | --- |
| MALARIA (ALL SPECIES) | 6147 | 18.06 | DIARRHEA, ACUTE UNSPECIFIED | 1068 | 20.64 | RESPIRATORY INFECTION, ACUTE | 610 | 13.89 | DIARRHEA, ACUTE UNSPECIFIED | 244 | 15.16 |
| DIARRHEA, ACUTE UNSPECIFIED | 3823 | 11.23 | DIARRHEA, CHRONIC | 554 | 10.71 | DIARRHEA, ACUTE UNSPECIFIED | 415 | 9.45 | RESPIRATORY INFECTION, ACUTE | 182 | 11.31 |
| RESPIRATORY INFECTION, ACUTE | 2425 | 7.13 | RABIES PEP (post exposure prophylaxis) | 332 | 6.42 | DIARRHEA ACUTE BACTERIAL | 259 | 5.90 | BITE, ANIMAL | 129 | 8.02 |
| VIRAL SYNDROME (WITH/WITHOUT RASH) | 2273 | 6.68 | BITE, ANIMAL | 322 | 6.22 | INSECT OR OTHER ARTHROPOD BITE/STING (WITH OR WITHOUT SUPRAINFECTION) | 231 | 5.26 | RABIES PEP (post exposure prophylaxis) | 120 | 7.46 |
| DIARRHEA, CHRONIC | 1580 | 4.64 | RESPIRATORY INFECTION, ACUTE | 256 | 4.95 | SKIN & SOFT TISSUE INFECTIONS | 212 | 4.83 | DIARRHEA ACUTE BACTERIAL | 90 | 5.59 |
| SCHISTOSOMIASIS | 1423 | 4.18 | DIARRHEA ACUTE BACTERIAL | 249 | 4.81 | AIDS, HIV, SYPHILIS, GONORRHEA | 183 | 4.17 | SKIN & SOFT TISSUE INFECTIONS | 50 | 3.11 |
| SKIN & SOFT TISSUE INFECTIONS | 1173 | 3.45 | DIARRHEA ACUTE parasitic | 181 | 3.50 | BITE, ANIMAL | 140 | 3.19 | DIARRHEA, CHRONIC | 44 | 2.73 |
| DIARRHEA ACUTE parasitic | 1151 | 3.38 | SKIN & SOFT TISSUE INFECTIONS | 147 | 2.84 | VIRAL SYNDROME (WITH/WITHOUT RASH) | 128 | 2.91 | INSECT OR OTHER ARTHROPOD BITE/STING (WITH OR WITHOUT SUPRAINFECTION) | 34 | 2.11 |
| DIARRHEA ACUTE BACTERIAL | 951 | 2.79 | INSECT OR OTHER ARTHROPOD BITE/STING (WITH OR WITHOUT SUPRAINFECTION) | 108 | 2.09 | DIARRHEA, CHRONIC | 125 | 2.85 | VIRAL SYNDROME (WITH/WITHOUT RASH) | 31 | 1.93 |
| BLASTOCYSTIS Sp. | 817 | 2.40 | ANXIETY/FATIGUE | 107 | 2.07 | RABIES PEP (post exposure prophylaxis) | 117 | 2.66 | AIDS, HIV, SYPHILIS, GONORRHEA | 28 | 1.74 |
| FEBRILE ILLNESS, UNSPECIFIED (< 3 WEEKS) | 782 | 2.30 | VIRAL SYNDROME (WITH/WITHOUT RASH) | 106 | 2.05 | LEISHMANIA | 94 | 2.14 | MYCOBACTERIUM TUBERCULOSIS | 28 | 1.74 |
| INSECT OR OTHER ARTHROPOD BITE/STING (WITH OR WITHOUT SUPRAINFECTION) | 738 | 2.17 | SCHISTOSOMIASIS | 94 | 1.82 | DIARRHEA ACUTE parasitic | 85 | 1.94 | DIARRHEA ACUTE parasitic | 26 | 1.62 |
| ANXIETY/FATIGUE | 694 | 2.04 | BLASTOCYSTIS Sp. | 93 | 1.80 | PYELONEPHRITIS | 54 | 1.23 | TRAUMA OR OVERUSE | 22 | 1.37 |
| RICKETTSIA | 681 | 2.00 | LEISHMANIA | 73 | 1.41 | BLASTOCYSTIS Sp. | 51 | 1.16 | HEPATITIS (VIRAL- CHRONIC) | 21 | 1.31 |
| NEMATODE INFECTIONS, INTESTINAL | 458 | 1.35 | HEPATITIS (VIRAL- ACUTE) | 72 | 1.39 | TRAUMA OR OVERUSE | 50 | 1.14 | HEPATITIS (VIRAL- ACUTE) | 20 | 1.24 |
| AIDS, HIV, SYPHILIS, GONORRHEA | 418 | 1.23 | FEBRILE ILLNESS, UNSPECIFIED (< 3 WEEKS) | 59 | 1.14 | MENINGITIS | 46 | 1.05 | ANXIETY/FATIGUE | 19 | 1.18 |
| URINARY TRACT INF, ACUTE | 358 | 1.05 | ABDOMINAL PAIN, UNSPECIFIED ETIOLOGY | 45 | 0.87 | URINARY TRACT INF, ACUTE | 46 | 1.05 | OTITIS MEDIA, ACUTE | 18 | 1.12 |
| CUTANEOUS LARVA MIGRANS, HOOKWORM-RELATED | 312 | 0.92 | MALARIA (ALL SPECIES) | 45 | 0.87 | ANXIETY/FATIGUE | 42 | 0.96 | URINARY TRACT INF, ACUTE | 15 | 0.93 |
| EOSINOPHILIA | 293 | 0.86 | EOSINOPHILIA | 42 | 0.81 | RASH, UNKNOWN ETIOLOGY (NON-FEBRILE) | 42 | 0.96 | BLASTOCYSTIS Sp. | 13 | 0.81 |
| FUNGAL INFECTION (SUPERFICIAL/SUBCUTANEOUS/CUTANEOUS MYCOSIS) | 287 | 0.84 | URINARY TRACT INF, ACUTE | 41 | 0.79 | FUNGAL INFECTION (SUPERFICIAL/SUBCUTANEOUS/CUTANEOUS MYCOSIS) | 41 | 0.93 | MENINGITIS | 13 | 0.81 |

| **Australia/New Zealand N=472** | **Freq** | **%** | **Middle East N=3120** | **Freq** | **%** | **Antarctica N=4** | **Freq** | **%** | **Plane Ship N=196** | **Freq** | **%** |
| --- | --- | --- | --- | --- | --- | --- | --- | --- | --- | --- | --- |
| INSECT OR OTHER ARTHROPOD BITE/STING (WITH OR WITHOUT SUPRAINFECTION) | 54 | 11.44 | RESPIRATORY INFECTION, ACUTE | 504 | 16.15 | BLASTOCYSTIS Sp. | 1 | 25 | RESPIRATORY INFECTION, ACUTE | 50 | 25.51 |
| SKIN & SOFT TISSUE INFECTIONS | 52 | 11.02 | DIARRHEA, ACUTE UNSPECIFIED | 486 | 15.58 | CIGUATERA INTOXICATION | 1 | 25 | DIARRHEA, ACUTE UNSPECIFIED | 22 | 11.22 |
| RESPIRATORY INFECTION, ACUTE | 36 | 7.63 | RABIES PEP (post exposure prophylaxis) | 202 | 6.47 | DIARRHEA ACUTE parasitic | 1 | 25 | VIRAL SYNDROME (WITH/WITHOUT RASH) | 15 | 7.65 |
| DIARRHEA, CHRONIC | 22 | 4.66 | BITE, ANIMAL | 194 | 6.22 | NEMATODE INFECTIONS, INTESTINAL | 1 | 25 | SKIN & SOFT TISSUE INFECTIONS | 10 | 5.10 |
| ARTHRALGIA/BONE PAIN | 21 | 4.45 | DIARRHEA, CHRONIC | 187 | 5.99 |  |  |  | DIARRHEA ACUTE BACTERIAL | 7 | 3.57 |
| VIRAL SYNDROME (WITH/WITHOUT RASH) | 19 | 4.03 | DIARRHEA ACUTE BACTERIAL | 185 | 5.93 |  |  |  | DIARRHEA, CHRONIC | 7 | 3.57 |
| DIARRHEA, ACUTE UNSPECIFIED | 18 | 3.81 | SKIN & SOFT TISSUE INFECTIONS | 99 | 3.17 |  |  |  | ANXIETY/FATIGUE | 4 | 2.04 |
| SELECTED VIRUSES (eg YELLOW FEVER, ROSS RIVER ETC) | 18 | 3.81 | DIARRHEA ACUTE parasitic | 85 | 2.72 |  |  |  | MALARIA (ALL SPECIES) | 4 | 2.04 |
| RASH, UNKNOWN ETIOLOGY (NON-FEBRILE) | 17 | 3.60 | VIRAL SYNDROME (WITH/WITHOUT RASH) | 80 | 2.56 |  |  |  | BLASTOCYSTIS Sp. | 3 | 1.53 |
| BLASTOCYSTIS Sp. | 10 | 2.12 | INSECT OR OTHER ARTHROPOD BITE/STING (WITH OR WITHOUT SUPRAINFECTION) | 62 | 1.99 |  |  |  | HEADACHE | 3 | 1.53 |
| ARTHRITIS, NONSEPTIC | 9 | 1.91 | BLASTOCYSTIS Sp. | 43 | 1.38 |  |  |  | HEART DISEASE | 3 | 1.53 |
| ABDOMINAL PAIN, UNSPECIFIED ETIOLOGY | 8 | 1.69 | ANXIETY/FATIGUE | 40 | 1.28 |  |  |  | HEPATITIS (VIRAL- CHRONIC) | 3 | 1.53 |
| FUNGAL INFECTION (SUPERFICIAL/SUBCUTANEOUS/CUTANEOUS MYCOSIS) | 8 | 1.69 | LEISHMANIA | 40 | 1.28 |  |  |  | INSECT OR OTHER ARTHROPOD BITE/STING (WITH OR WITHOUT SUPRAINFECTION) | 3 | 1.53 |
| BITE, TICK | 7 | 1.48 | NEMATODE INFECTIONS, INTESTINAL | 32 | 1.03 |  |  |  | PULMONARY EMBOLISM | 3 | 1.53 |
| DIARRHEA ACUTE parasitic | 7 | 1.48 | AIDS, HIV, SYPHILIS, GONORRHEA | 26 | 0.83 |  |  |  | SEPSIS | 3 | 1.53 |
| HEADACHE | 7 | 1.48 | RASH, UNKNOWN ETIOLOGY (NON-FEBRILE) | 26 | 0.83 |  |  |  | TRAUMA OR OVERUSE | 3 | 1.53 |
| ANXIETY/FATIGUE | 6 | 1.27 | URINARY TRACT INF, ACUTE | 26 | 0.83 |  |  |  | AIDS, HIV, SYPHILIS, GONORRHEA | 2 | 1.02 |
| DIARRHEA ACUTE BACTERIAL | 6 | 1.27 | ABDOMINAL PAIN, UNSPECIFIED ETIOLOGY | 24 | 0.77 |  |  |  | ARTHRALGIA/BONE PAIN | 2 | 1.02 |
| AIDS, HIV, SYPHILIS, GONORRHEA | 5 | 1.06 | HEPATITIS (VIRAL- ACUTE) | 21 | 0.67 |  |  |  | BITE, ANIMAL | 2 | 1.02 |
| NEMATODE INFECTIONS, INTESTINAL | 5 | 1.06 | FEBRILE ILLNESS, UNSPECIFIED (FEVER OF UNKNOWN ORIGIN) (>=3 WEEKS) | 19 | 0.61 |  |  |  | DENGUE, UNCOMPLICATED | 2 | 1.02 |

**Supplementary Table 3a.** Death records in various GeoSentinel/EuroTravNet sentinel surveillance (sub)cohorts. Note that there is a partial cohort overlap between the various analyses, depending on reporting period, (sub)cohort and analytical approach chosen.

| Authors | Years included | % deaths | N | inclusions | Sites |
| --- | --- | --- | --- | --- | --- |
| Leder et al. 2013^12^ | 2007-2011 | 0.066% | 28/42,173 | Seen after travel | All |
| Jensenius et al. 2013 | 1996-2011 | 0.356% | 13/3,655 | Seen during or after travel, living in higher developed countries, visiting lesser developed countries, only with life threatening illness | All |
| Schlagenhauf et al. 2015^10^ | 2008-2012 | 0.034% | 11/32,136 | After or during travel, | EuroTravNet |
| Hagman et al. 2014 | 2000-2012 | 0.031% | 3/9,624 | After travel only | US clinics |
| Chen et al. 2018 | 1997-2014 | 0.107% | 13/12,203 | Business travelers | All |
| Harveyet al. 2013 | 1997-2011 | 0.023% | 3/13,059 | After travel | US clinics |
| Data reported here | 1998-2018 | 0.098% | 11/11,239 | Migrant travel only | EuroTravNet |
| Data reported here | 1998-2018 | 0.037% | 34/92,500 | After and during travel | EuroTravNet |
| Data reported here | 1998-2018 |  | 45/103,739 | All | EuroTravNet |

References:

Chen LH, Leder K, Barbre KA, et al. Business travel-associated illness: a GeoSentinel analysis. J Travel Med 2018;25.

Hagmann SH, Han PV, Stauffer WM, et al. Travel-associated disease among US residents visiting US GeoSentinel clinicsafter return from international travel. Fam Pract. 2014 ;31(6):678-87.

Harvey K, Esposito DH, Han P, et al. Surveillance for travel-related disease—GeoSentinel Surveillance System, United States, 1997-20111. MMWR Surveill Summ 2013;62:1-23.

Jensenius M, Han PV, Schlagenhauf P, et al. Acute and potentially life-threatening tropical diseases in western travelers – a GeoSentinel multicenter study, 1996-2011. Am J Trop Med Hyg 2013;88:397-404.

Leder K, Torresi J, Libman MD, et al. GeoSentinel surveillance of illness in returned travelers, 2007-2011. Ann Intern Med 2013;158(6):456-68.^10^

Schlagenhauf P, Weld L, Goorhuis A, et al. Travel-associated infection presenting in Europe (2008-12): an analysis of EuroTravNet longitudinal surveillance data, and evaluation of the effect of the pre-travel consultation. Lancet Infect Dis 2015;15(1):55-64.^9^

**Supplementary Table 3b**: Causes of death in 45 patients in our cohort described here.

| **Diagnoses** | **Age** | **Sex** | **Region of exposure** | **Reason for Travel** |
| --- | --- | --- | --- | --- |
| ARDS, AIDS | 28 | Female | Caribbean | Tourism |
| Melioidosis | 35 | Male | Caribbean | Tourism |
| Fungal pneumonia | 40 | Female | Caribbean | Migration |
| Multi organ failure due to Salmonella species, sepsis | 55 | Male | Caribbean | Tourism |
| Pneumococcal meningitis | 64 | Female | Eastern Europe | VFR |
| Atypical/non-lobar pneumonia, Legionnaires' disease, acute renal failure | 65 | Male | Eastern Europe | Tourism |
| TB | 82 | Male | Eastern Europe | Migration |
| Pneumococcal pneumonia | 77 | Male | Middle East | Tourism |
| Pulmonary TB Disseminated/miliary TB, CNS tuberculoma | 77 | Female | Middle East | Migration |
| Pyogenic liver abscess, diabetes mellitus | 34 | Female | Not Ascertainable | Migration |
| Asymptomatic HIV, TB | 37 | Female | Not Ascertainable | VFR |
| Acute hepatic insufficiency after NSAID overdose | 46 | Male | Not Ascertainable | Tourism |
| Pulmonary embolism | 52 | Female | Not Ascertainable | Tourism |
| Cytomegalovirus, AIDS | 55 | Male | Not Ascertainable | Migration |
| Lobar pneumonia | 84 | Male | PlaneShip | Tourism |
| Yellow fever | 43 | Male | South America | Tourism |
| Extrapulmonary TB | 40 | Male | South Central Asia | Migration |
| Lobar pneumonia, Sepsis | 75 | Male | South Central Asia | VFR |
| Arterial dissection | 38 | Male | South East Asia | Business |
| HIV, acute infection (febrile), autoimmune disorders | 47 | Male | South East Asia | Tourism |
| Melioidosis^1^ | 53 | Male | South East Asia | Tourism |
| Dengue DSS | 53 | Female | South East Asia | Tourism |
| *Naegleria fowleri* encephalitis | 72 | Female | South East Asia | Tourism |
| Severe and complicated malaria | 26 | Male | Sub-Saharan Africa | Military |
| Visceral Leishmaniasis, chronic Hepatitis B, sepsis | 32 | Male | Sub-Saharan Africa | Migration |
| TB, AIDS | 33 | Female | Sub-Saharan Africa | Migration |
| AIDS | 34 | Male | Sub-Saharan Africa | Migration |
| Severe and complicated malaria | 40 | Male | Sub-Saharan Africa | Business |
| AIDS, lobar pneumonia, sepsis | 42 | Female | Sub-Saharan Africa | Migration |
| Extrapulmonary TB, pulmonary TB | 47 | Male | Sub-Saharan Africa | VFR |
| *P. falciparum* malaria | 48 | Male | Sub-Saharan Africa | VFR |
| *P. falciparum* malaria, severe and complicated | 53 | Male | Sub-Saharan Africa | Business |
| *P. falciparum* malaria, severe and complicated | 55 | Female | Sub-Saharan Africa | Business |
| Severe and complicated malaria | 57 | Male | Sub-Saharan Africa | M/V/R/A |
| Granulomatous Amoebic Encephalitis (GAE) | 60 | Female | Sub-Saharan Africa | Tourism |
| Fungal pneumonia, sepsis | 61 | Female | Sub-Saharan Africa | Migration |
| Severe and complicated malaria | 68 | Male | Sub-Saharan Africa | Tourism |
| Viral syndrome (with/without rash) | 6 | Male | Western Europe | Tourism |
| Acute liver failure, leishmaniasis | 56 | Male | Western Europe | Tourism |
| Visceral leishmaniasis | 59 | Male | Western Europe | Tourism |
| Pneumococcal meningitis | 65 | Female | Western Europe | Tourism |
| Pyelonephritis, sepsis | 67 | Male | Western Europe | Tourism |
| Acute UTI, sepsis | 78 | Female | Western Europe | Tourism |
| ARDS, lobar pneumonia, sepsis | 84 | Female | Western Europe | Tourism |
| Lobar pneumonia, COPD, Parkinson’s disease | 86 | Male | Western Europe | Tourism |

AIDS = acquired immunodeficiency syndrome; ARDS = Acute respiratory distress syndrome; CNS = central nervous system; COPD = chronic obstructive pulmonary disease; DSS = dengue shock syndrome; HIV = human immunodeficiency virus; MTB = *Mycobacerium tuberculosis*; NSAID = non-steroidal anti-inflammatory drugs; UTI = urinary tract infection

^1^This patient who died of melioidosis was diagnosed and cared for in Switzerland while transiting to his home country.

**Supplementary Table 4:** Rare diagnoses

| \| Diagnosis \| 1998-2002 \| 2003-2007 \| 2008-2012 \| 2013-2018 \| total \| \| --- \| --- \| --- \| --- \| --- \| --- \| \| BARTONELLA HENSELAE (OTHER THAN CAT SCRATCH DISEASE) \| 1 \| 1 \| 2 \| 3 \| 7 \| \| BRUCELLOSIS, ACUTE \| 2 \| 3 \| 16 \| 17 \| 38 \| \| CAT SCRATCH DISEASE (BARTONELLA HENSELAE) \| 0 \| 0 \| 3 \| 7 \| 10 \| \| DENGUE, COMPLICATED \| 0 \| 1 \| 9 \| 19 \| 29 \| \| E. COLI, SHIGA TOXIN PRODUCING (aka Enterohemorrhagic E. COLI, EHEC/Verocytotoxin-producing E. coli, VTEC) includes E. coli 0157:H7* \| 0 \| 0 \| 13 \| 35 \| 48 \| \| EBOLA VIRUS \| 0 \| 0 \| 0 \| 3 \| 3 \| \| ECHINOCOCCOSIS, HEPATIC* \| 4 \| 14 \| 71 \| 109 \| 198 \| \| ECHINOCOCCOSIS, HEPATIC and NON-HEPATIC \| 2 \| 2 \| 15 \| 20 \| 39 \| \| ECHINOCOCCOSIS, NON-HEPATIC \| 7 \| 6 \| 20 \| 24 \| 57 \| \| ENCEPHALITIS, ACUTE SPECIFIC ETIOLOGY \| 0 \| 2 \| 4 \| 12 \| 18 \| \| ENCEPHALITIS, JAPANESE \| 0 \| 0 \| 1 \| 4 \| 5 \| \| ENCEPHALITIS, MURRAY VALLEY \| 0 \| 0 \| 0 \| 1 \| 1 \| \| ENCEPHALITIS, TICK BORNE \| 0 \| 5 \| 4 \| 4 \| 13 \| \| GNATHOSTOMA \| 5 \| 5 \| 13 \| 8 \| 31 \| \| HANTAVIRUS \| 0 \| 0 \| 0 \| 2 \| 2 \| \| HISTOPLASMOSIS \| 0 \| 3 \| 7 \| 14 \| 24 \| \| LEGIONNAIRES' DISEASE* \| 0 \| 5 \| 27 \| 51 \| 83 \| \| LEPTOSPIROSIS* \| 2 \| 4 \| 56 \| 130 \| 192 \| \| MELIOIDOSIS \| 1 \| 0 \| 7 \| 12 \| 20 \| \| MENINGITIS, MENINGOCOCCAL \| 0 \| 3 \| 3 \| 3 \| 9 \| \| NEUROCYSTICERCOSIS \| 3 \| 8 \| 38 \| 50 \| 99 \| \| Q FEVER (COXIELLA BURNETII) \| 0 \| 4 \| 26 \| 22 \| 52 \| \| RIFT VALLEY FEVER \| 0 \| 0 \| 2 \| 1 \| 3 \| \| ROSS RIVER VIRUS \| 0 \| 0 \| 5 \| 14 \| 19 \| \| RUBELLA \| 3 \| 1 \| 8 \| 2 \| 14 \| \| SARCOCYSTOSIS, MUSCULAR \| 0 \| 0 \| 18 \| 11 \| 29 \| \| TRYPANOSOMIASIS, AFRICAN (T. B. GAMBIENSE) \| 0 \| 0 \| 1 \| 0 \| 1 \| \| TRYPANOSOMIASIS, AFRICAN (T. B. RHODESIENSE) \| 0 \| 0 \| 3 \| 3 \| 6 \| \| TULAREMIA \| 0 \| 1 \| 0 \| 3 \| 4 \| \| WEST NILE VIRUS \| 0 \| 1 \| 3 \| 3 \| 7 \| \| YELLOW FEVER \| 0 \| 0 \| 0 \| 5 \| 5 \| \|  \| 30 \| 69 \| 375 \| 592 \| 1066 \| |
| --- | --- | --- | --- | --- | --- | --- | --- | --- | --- | --- | --- | --- | --- | --- | --- | --- | --- | --- | --- | --- | --- | --- | --- | --- | --- | --- | --- | --- | --- | --- | --- | --- | --- | --- | --- | --- | --- | --- | --- | --- | --- | --- | --- | --- | --- | --- | --- | --- | --- | --- | --- | --- | --- | --- | --- | --- | --- | --- | --- | --- | --- | --- | --- | --- | --- | --- | --- | --- | --- | --- | --- | --- | --- | --- | --- | --- | --- | --- | --- | --- | --- | --- | --- | --- | --- | --- | --- | --- | --- | --- | --- | --- | --- | --- | --- | --- | --- | --- | --- | --- | --- | --- | --- | --- | --- | --- | --- | --- | --- | --- | --- | --- | --- | --- | --- | --- | --- | --- | --- | --- | --- | --- | --- | --- | --- | --- | --- | --- | --- | --- | --- | --- | --- | --- | --- | --- | --- | --- | --- | --- | --- | --- | --- | --- | --- | --- | --- | --- | --- | --- | --- | --- | --- | --- | --- | --- | --- | --- | --- | --- | --- | --- | --- | --- | --- | --- | --- | --- | --- | --- | --- | --- | --- | --- | --- | --- | --- | --- | --- | --- | --- | --- | --- | --- | --- | --- | --- | --- | --- | --- | --- | --- | --- | --- | --- | --- | --- | --- |

*Significant increase or decrease in % of patients over time

**Supplementary Figure 1a-d.** Group characteristics changes over time by year group.

**Supplementary Figure 1a.** Age Group by year group.

**
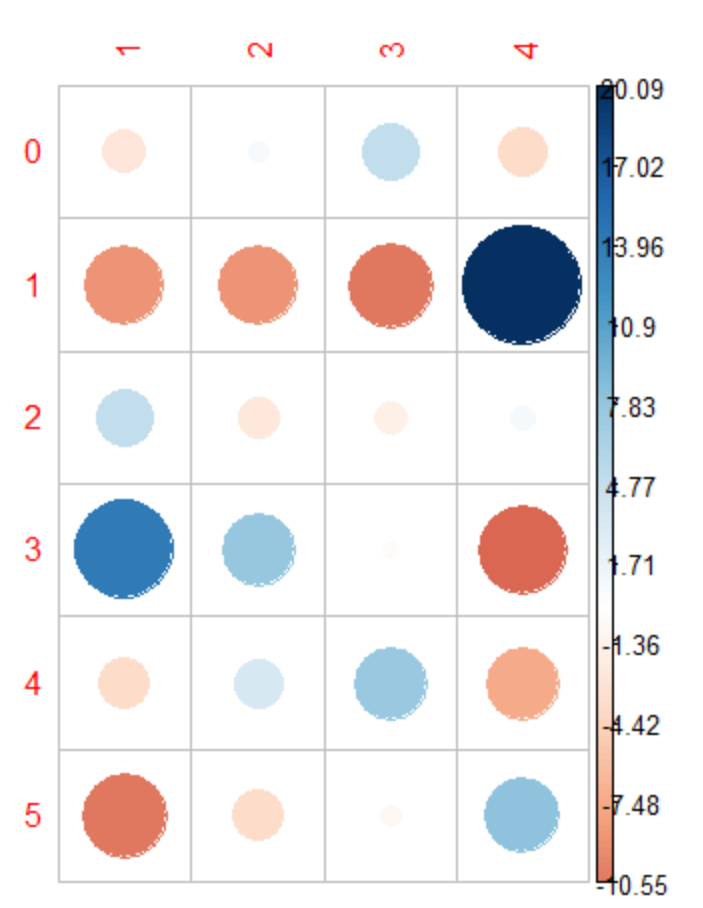
**

Columns are the 4 year groups. The rows are 0=age newborn to 10, 1=10-20, 2=20-30, 3=30-40, 4=40-50, 5=50+ years old. The bigger the dots, the bigger is the residual and the bigger the contribution to the chi-square. Blue is positive, brick is negative. So the biggest pattern is that % of children age 10-20 increased from orange to blue, the colours are strongest and the size of the dots the biggest. Then the decrease from blue to orange for 30-40 year olds is the next biggest contributor to the significant result and finally the increase from orange to blue for those over 50. We combined the last age groups because the numbers were small and it meant the results were unstable.

**Supplementary Figure 1b.** Sex by year group.


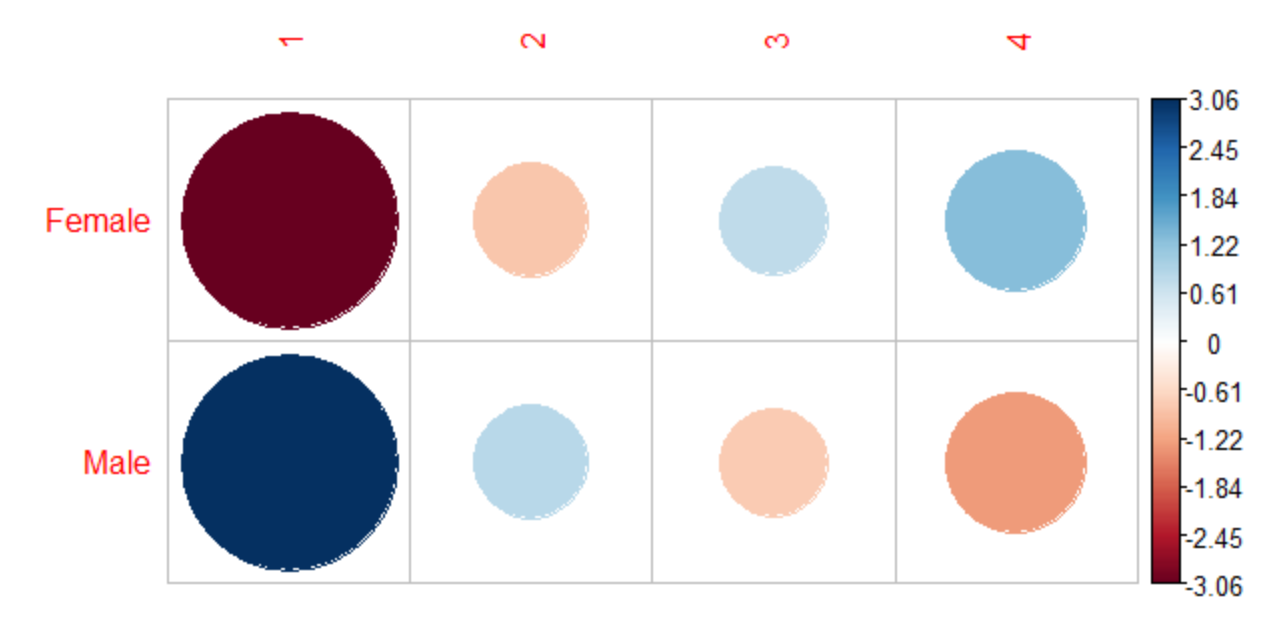


Columns are the 4 year groups. The bigger the dots, the bigger is the residual and the bigger the contribution to the chi-square. Blue is positive, brick is negative.

**Supplementary Figure 1c.** Changes in traveller groups over time.


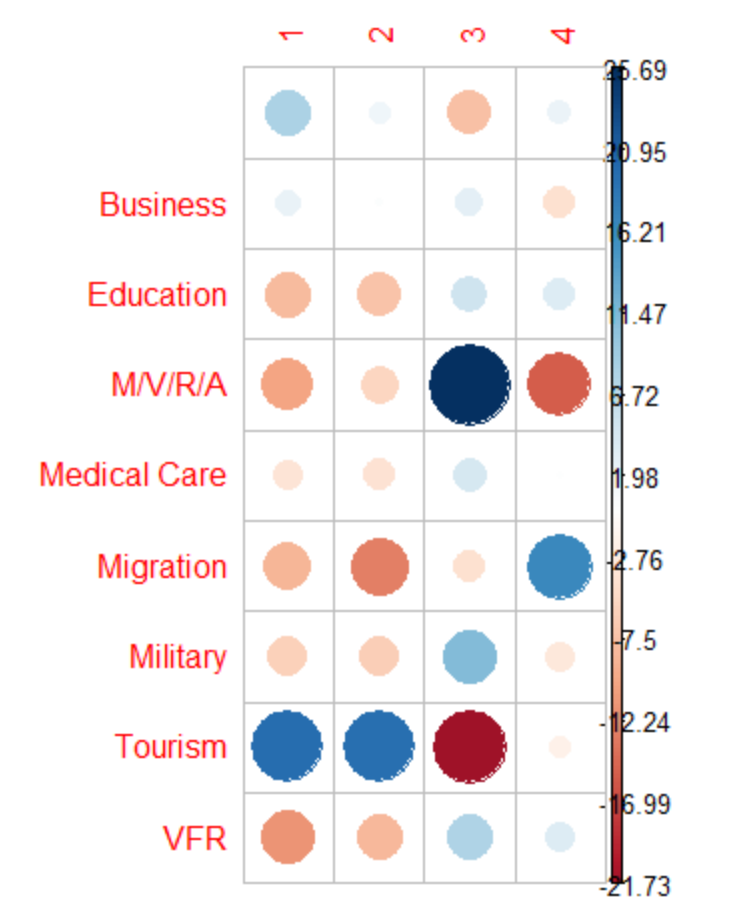


Columns are the 4 year groups. The bigger the dots, the bigger is the residual and the bigger the contribution to the chi-square. Blue is positive, brick is negative.

**Supplementary Figure 1d.**


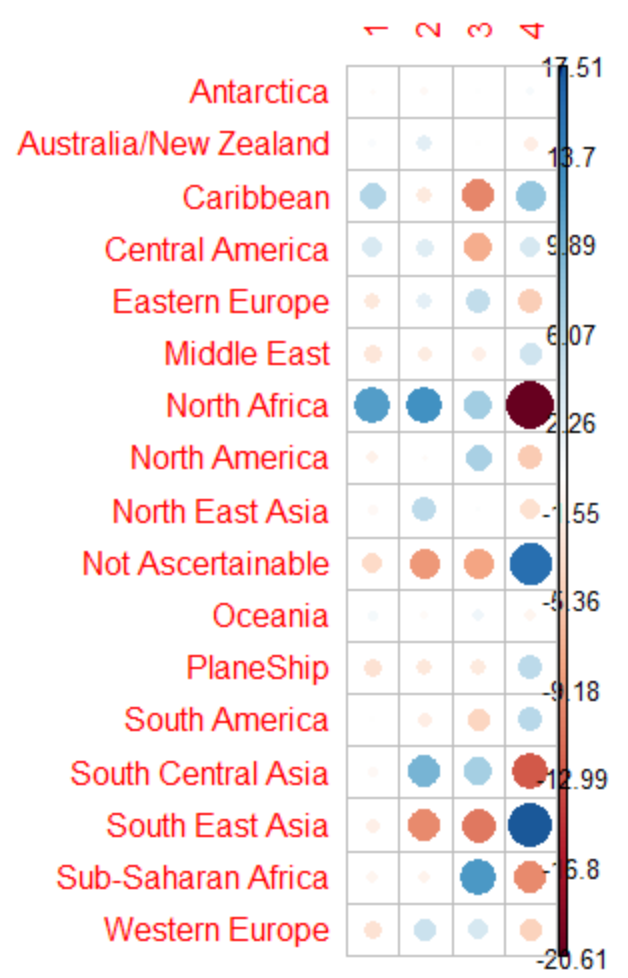


Columns are the 4 year groups. The bigger the dots, the bigger is the residual and the bigger the contribution to the chi-square. Blue is positive, brick is negative.

**Supplementary Figure 2.** Viral haemorrhagic fevers in patients seen during or after travel, N=92,500.


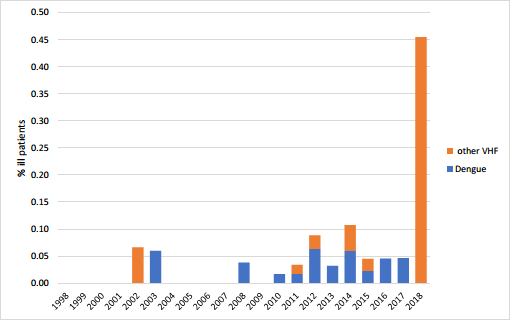


Diagnoses of dengue fever that were not complicated were not included in this table. complicated dengue (DHF, DSS) = 28; yellow fever = 5; Rift Valley fever = 3; Ebola = 3; hantavirus disease (unspecified) = 2; Crimean-Congo fever = 1; Lassa fever = 1.

**Supplementary Figure 3.** Vaccine preventable diseases in patients seen during or after travel.


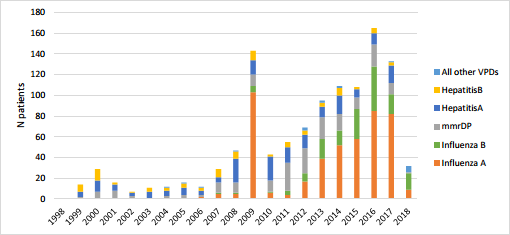


VPDs = vaccine-preventable diseases;

mmrDP = measles, mumps, rubella, diphteria, polio
